# Exposure to concentrated ambient fine particulate matter disrupts vascular endothelial cell barrier function via the IL‐6/HIF‐1α signaling pathway

**DOI:** 10.1002/2211-5463.12077

**Published:** 2016-05-24

**Authors:** Jianwei Dai, Canxing Sun, Zhuo Yao, Wensheng Chen, Lihong Yu, Minhui Long

**Affiliations:** ^1^ Department of BiotechnologyGuangzhou Medical UniversityChina; ^2^ Tianjin University of Science & TechnologyChina

**Keywords:** fine particulate matter, HIF‐1α, IL‐6, permeability, vascular endothelial cell

## Abstract

Exposure to concentrated ambient fine particulate matter (PM2.5) has been associated with cardiovascular diseases (CVDs). The barrier function of vascular endothelial cells is critical for the development of CVDs. Here, we employed human umbilical vein endothelial cells to clarify the function of ambient PM2.5 pollution in the regulation of membrane permeability of vascular endothelial cells. The results show that a high concentration of PM2.5, which mainly includes heavy metals and polycyclic aromatic hydrocarbons, induces barrier dysfunction of vascular endothelial cells. This was mediated in part by promoting IL‐6 expression, which then increases the transcriptional activity of HIF‐1α by promoting its translocation to the nucleus. Our findings indicate that concentrated PM2.5 can destroy membrane integrity and promote permeability in vascular endothelial cells, thereby contributing to the development of CVDs.

**Abbreviation**
AS: atherosclerosis
CCK‐8: Cell Counting Kit‐8
CVDs: cardiovascular diseases
EGM: endothelial cell growth medium
EVCs: early vascular cells
FITC: fluorescein isothiocyanate
GM‐CSF: granulocyte‐macrophage colony stimulating factor
HIF‐1α: hypoxia‐inducible factor‐1α
HIF: hypoxia‐inducible factor
HUVECs: human umbilical vein endothelial cells
MLCK: myosin light chain kinase
PAHs: polycyclic aromatic hydrocarbons
PM2.5: ambient fine particulate matter
PMSF: phenylmethylsulfonyl fluoride
RIPA: radio‐immunoprecipitation assay
ROS: reactive oxygen species
TNF‐α: tumor necrosis factor‐α
TRPM2: transient receptor potential channels
VECs: vascular endothelium cells
ZO‐1: zonula occludens‐1

There is increasing concern about the potential deleterious effects of ambient air pollution, which is currently recognized as a significant risk to global public health. A growing body of epidemiological and clinical research data have demonstrated that short‐ and long‐term exposure to ambient particulate matter less than 2.5 μm in aerodynamic diameter (PM2.5, fine particles) is linked to an increase in cardiovascular disease (CVD), morbidity and mortality, particularly in accelerating atherosclerosis (AS) development 1, 2, 3, 4, 5. Several recent reports provide experimental evidence that demonstrates that the translocation of PM2.5 from the lung and directly into the blood exacerbates the progression of AS by initiating acute inflammatory responses 6, 7, 8. However, the underlying pathophysiologic mechanisms that link PM2.5 exposure to AS remain elusive.

Atherosclerosis is a disease that involves the arteries and is recognized as the primary cause of CVDs, particularly in westernized societies. Research investigations have indicated that the elevated permeability of the vascular endothelium, which is the innermost layer of the artery, is a key early event in the formation of AS lesions 9, 10, 11. The endothelium is the major constituent of the vascular wall, which acts as a selective barrier to control permeability of plasma components. An increase in the permeability of endothelial cells generally results in the accumulation of fat and cholesterol within the arterial walls. This in turn induces plaque buildup, which causes shrinkage of the diameter of arteries, thereby impeding blood flow and ultimately resulting in oxygen shortage. During AS progression, an enlarged plaque may break off into smaller pieces, thus causing the formation of a blood clot, which can block arteries, cut off its blood supply, and cause life‐threatening cardiovascular events. Therefore, endothelial permeability is critical for the formation and development of AS.

Earlier studies have indicated that hypoxia and inflammation can increase endothelial permeability 12, 13, 14. Therefore, the level of hypoxia‐inducible factors (HIFs) is often elevated in patients with inflammatory disorders 15, 16. It has been previously reported that PM2.5 induces cardiac hypoxia 17. However, whether exposure to PM2.5 results in the induction of hypoxia in endothelial cells remains unknown. This has therefore prompted us to hypothesize that exposure to PM2.5 promotes inflammation that induces hypoxia in vascular endothelium cells (VECs), which in turn increases the permeability of the endothelium.

In this study, we used VECs to define the possible role and the underlying mechanism of PM2.5 in cellular injury.

## Materials and methods

### Regents and antibodies

DAPI (4′,6‐diamidino‐2‐phenylindole) (KGR0001) was purchased from KeyGen Biotech (Nanjing, China). TRIzol was obtained from Invitrogen (CA, USA). Recombinant human IL‐6 (I1395) was obtained from Sigma‐Aldrich (St. Louis, MO, USA). The siRNA duplex for HIF‐1α was synthesized by Genepharma Co., Ltd. (Shanghai, China), as described elsewhere 18. Cell Counting Kit‐8 (C0037) was obtained from Beyotime (Jiangsu, China). Mouse IL‐6 enzyme‐linked immunosorbent assay (ELISA) Kit (EK0411) was obtained from Boster (Wuhan, China). The following primary antibodies were used for western blotting assay: rabbit anti‐HIF‐1α (PB0245, diluted to 1:500, Boster) and rabbit anti‐GAPDH (2118s, diluted to 1:2000, Cell Signaling Technology, Inc., Danvers, MA, USA).

### PM2.5 collection

Ambient PM2.5 samples were collected in Beijing on each day of measurement from November 2009 to June 2010 using IMPROVE Version II Samplers (URG; 22.8 L·min^−1^ flow rate) equipped with Teflo filters (25‐mm diameter, pore size: 3 μm, Pall, Port Washington, NY, USA). During the sampling period, three replicate ambient PM2.5 samples were consecutively collected for three days of each month on the same day (starting at 7:00 A.M.). The filters were removed from the samplers, and the particulates were pooled and lyophilized in a vacuum. The PM2.5 was dissolved in PBS (pH 7.2) and homogenized by ultrashaking for 15 min before treatment. The receptor source apportionment model of chemical mass balance (US EPA CMB8.2) was used to apportion sources of PM2.5 as previously described 19.

### Cell culture

Human umbilical vein endothelial cells (HUVECs) were obtained from the American Type Culture Collection (ATCC, VA, USA) and cultured in EGM endothelial cell growth medium (CC‐3121) with supplements (CC‐3124), which were purchased from Lonza (MD, USA). Cells were incubated at 37 °C in a humidified chamber containing 5% CO_2_. Lipofectamin 3000 (Invitrogen, Carlsbad CA, USA) was used to transfect siRNA at a final concentration of 100 nm.

### Analysis of cell viability

The HUVECs were plated at density of 2000 cells/well in 96‐well plates. Then, the indicated concentration of PM2.5 was added 24 h later and incubated at 37 °C for 24 h. Cell viability was evaluated using the Cell Counting Kit‐8. The data were obtained from three independent experiments. The percentage of viability was calculated using the following equation:

$$\text{Viability}\left(\%\right)=\left({\text{OD}}_{\mathrm{control}}-{\text{OD}}_{\mathrm{treatment}}\right)/{\text{OD}}_{\mathrm{control}}\times 100\%.$$

### Vascular permeability assay

Human umbilical vein endothelial cell monolayers were plated on the Transwell^™^ (Corning, Inc., Corning, NY, USA) insert and cultured until confluent. The chambers were washed with hydroxyethyl piperazine ethanesulfonic acid (HEPES) medium (endothelial cell growth medium), and FITC‐dextran (Invitrogen, Carlsbad, CA, USA) was added to the top chamber. After 24 h, the samples were removed from the bottom chamber and its absorbance was measured using a fluorometer (Berthold, Germany) at an excitation wavelength of 485 nm and an emission wavelength of 520 nm. The data were expressed as the mean of three experiments.

### Cytokine quantitation

The HUVECs were treated with various concentrations of PM2.5 (0, 40, and 80 μg·mL^−1^). Then, the cell culture media and the total protein of the cells (collected using RIPA lysis buffer) were collected for cytokine detection. Cytokine expression was detected by conducting a Bio‐Plex Pro^™^ Human Cytokine 8‐plex Assay (#M50‐000007A, Bio‐Rad, Hercules, CA, USA) using both cell culture medium and cell lysate.

### Quantitative real‐time PCR

Total RNA was extracted from treated cells using TRIzol, following the manufacturer's instructions. Total RNA (500 ng) was reverse transcribed using a PrimeScript^™^ RT Reagent Kit (RR037A, Takara, Japan), and relative quantity analysis of cytokines was conducted using SYBR^®^ Premix Ex Taq^™^ (Tli RNase H Plus) (RR037A, Takara, Japan). All the experiments were performed following the manufacturer's guidelines. β‐actin was used as loading control. All the primers (Table S1) were synthesized by Sangon Biotech (Shanghai, China).

### Western blot analysis

The total proteins from cells were prepared using RIPA lysis buffer, and 100 mm PMSF (Sigma) was added according to the manufacturer's recommendation. Proteins were separated by 10% SDS/PAGE, and then transferred to a polyvinylidene fluoride (PVDF) membrane (Millipore, Bedford, MA, USA). The membrane was washed with TBST [10 mm Tris‐HCl (pH 7.6), 150 mm NaCl, 0.05% Tween‐20], blocked with 1% BSA for 1 h, and then incubated with the appropriate primary antibodies overnight at 4 °C. The membrane was washed and then incubated with horseradish peroxide (HRP)‐labeled secondary antibody for 1 h at room temperature. Bands were visualized with an ECL Western Blot Detection System (Cell Signaling Technology, Beverly, MA, USA). Images of the bands were captured using the Molecular Image^®^ ChemiDoc^™^ XRS^+^with image lab ^™^ Software (Bio‐Rad Laboratories, Inc.). GAPDH was used as loading control.

### Immunofluorescence assay

The HUVECs (4 × 10^5^ cells/well) were plated in 6‐well plates and treated with PM2.5 at the concentration of 80 μg·mL^−1^. At 24 h post‐treatment, the cells were fixed for 20 min with 4% paraformaldehyde and incubated in 0.2% Triton‐X 100 for 15 min to allow permeabilization. Then, the cells were incubated with the corresponding primary antibody overnight at 4 °C, washed in PBS, and then incubated with fluorescently labeled secondary antibody for 1 h at room temperature. The fluorescent signal was viewed under a fluorescent microscope (Ti‐S, Nikon, Japan).

### Enzyme‐linked immunosorbent assay

The cell culture media of the NC group and PM2.5 group were collected, and the levels of IL‐6 were analyzed using human IL‐6 ELISA Kit (EK0410, Boster) according to the manufacturer's instructions. Each sample was assayed in three independent experiments.

### Statistical analyses

All data were expressed as the mean ± SD. Significant differences among individual groups were identified by ANOVA. All tests were performed using SPSS 19 (IBM Corp, Armonk, NY, USA), and *P* < 0.05 was considered statistically significant.

## Results

### PM2.5 inhibits vascular endothelial cell viability

**Figure 1:**
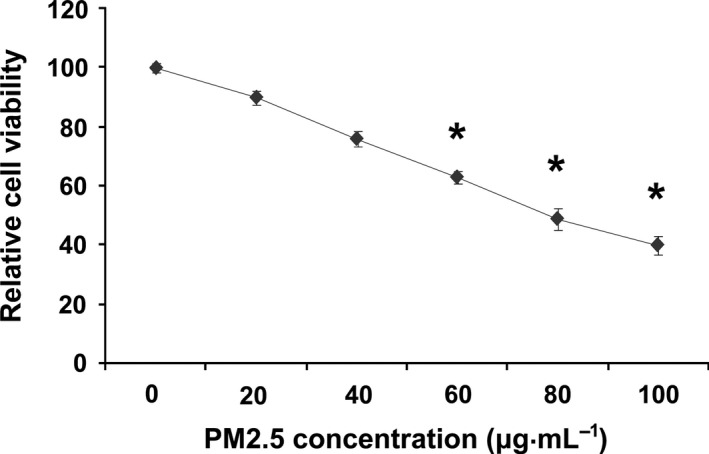
Ambient fine particulate matter inhibits HUVEC cell viability. The cells were treated with various doses of PM2.5 for 24 h, and cell viability was quantified using the CCk‐8 assay. The results represent three independent experiments. *P < 0.05.

Ten potential sources of PM2.5 from Beijing were identified using metal and organic tracers (Table 1). These sources were mainly composed of heavy metals, including aluminum (Al, 23–37%), iron (Fe, 28–29%), calcium (Ca, 13–17%), and zinc (Zn, 0.17–4%), and polycyclic aromatic hydrocarbons [PAHs (5, 6), 16–31%]. The proliferation of vascular endothelial cells (VECs) plays an important role in the development of AS. However, PM2.5 toxicity in VECs via blood circulation is unknown. To evaluate cellular toxicity of PM2.5 on VECs, the indicated concentration of PM2.5 was exposed to precultured HUVEC cells for 24 h. Figure 1 shows that PM2.5 markedly inhibited HUVEC viability in a dose‐dependent manner and showed antiproliferation activity, with an IC_50_ value of 80 μg·mL^−1^. However, whether PM2.5 affects the function of VECs was unknown.

**Table 1:** Relative abundance of pollutants in PM2.5 from Beijing

Pollutant	Min. concentration (%)	Max. concentration (%)
Aluminum	23	37
Calcium	13	17
Iron	28	29
Arsenic	0.015	0.02
Zinc	0.17	4
Copper	0.15	0.25
Cadmium	0.005	0.12
Lead	0.17	0.5
Polycyclic aromatic hydrocarbon PAH (4)	0.015	0.02
Polycyclic aromatic hydrocarbon PAH (5,6)	16	31

Analysis of PM2.5 indicted that the most abundant components were Al (23–37%), PAH (5,6) (16–31%), Fe (28–29%), Ca (13–17%) and Zn (0.17–4%). Others were present at less than 1%. Ambient PM2.5 samples were collected in Beijing on each day of measurement from November 2009 to June 2010.

### PM2.5 causes endothelial permeability

**Figure 2:**
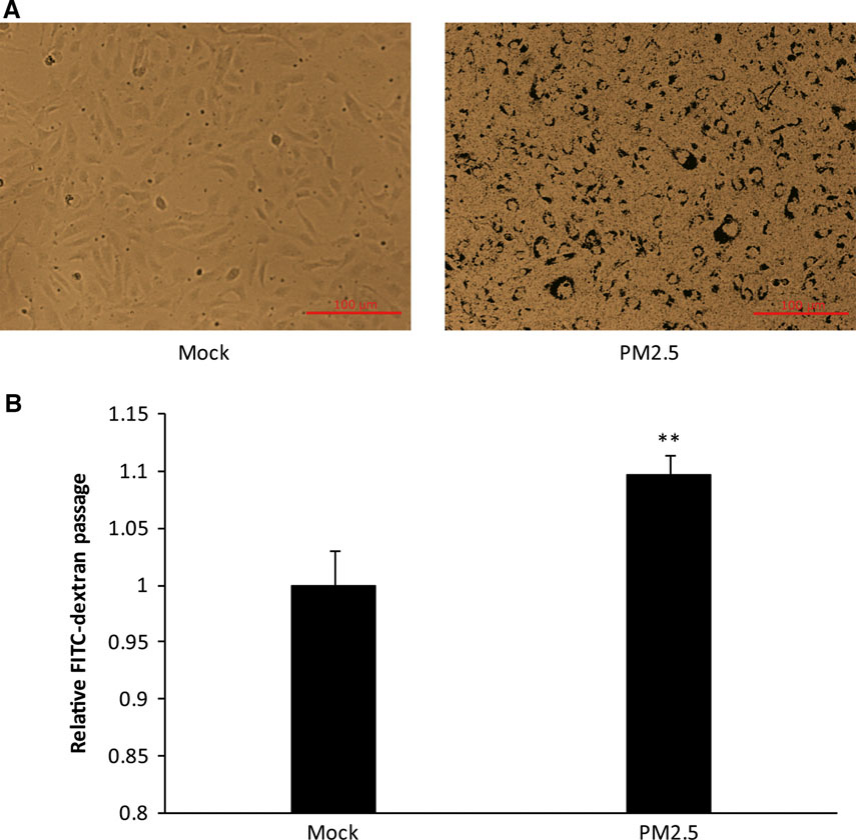
Ambient fine particulate matter destroys membrane integrity and increases permeability in HUVECs. (A) Brightfield microscopy showing monolayers of confluent endothelia (no PM2.5) and cells treated with PM2.5. (B) Effects of PM2.5 on the permeability of a HUVEC monolayer. Cells were stimulated with PBS (mock), PM2.5 80 μg·mL^−1^ for 24 h. **P < 0.01 Bar: 100 μm.

Experiments evaluating whether PM2.5 induces endothelial permeability were first performed using confluent monolayers of cells that were exposed to PM2.5 for 24 h. Figure 2A shows that the confluency rate of the cultured cells decreased. FITC‐dextran was employed to further confirm endothelial cell permeability after PM2.5 exposure. When HUVEC monolayers on culture inserts were treated with PM2.5, endothelial cell permeability, which was measured by the diffusion of FITC‐dextran across the HUVEC sheet, significantly increased compared to that observed in untreated control cultures (Fig. 2B).

### PM2.5 exposure promotes the secretion of IL‐6 in VECs

**Figure 3:**
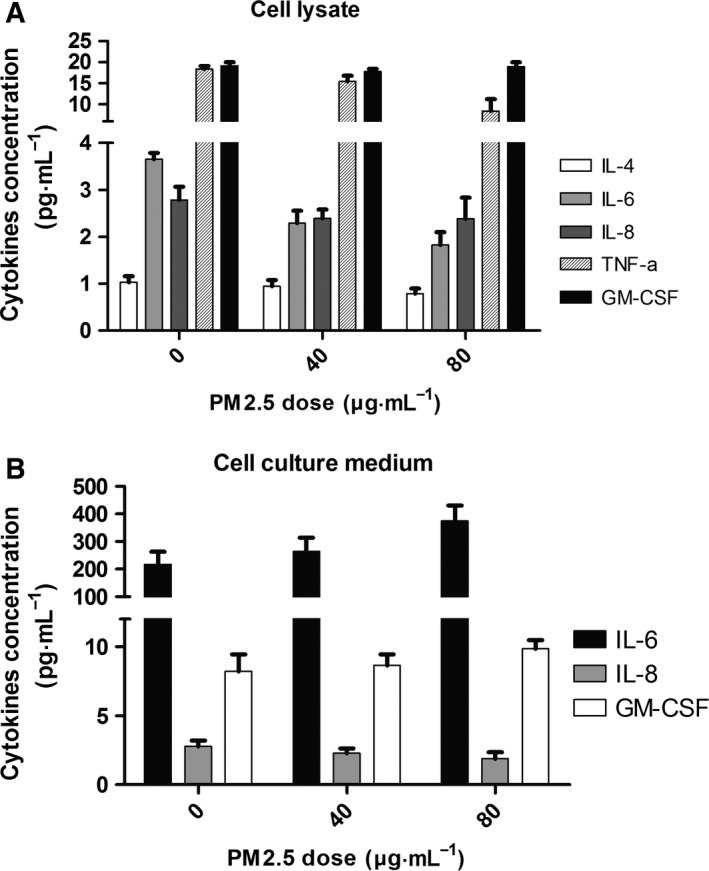
Ambient fine particulate matter promotes the secretion of IL‐6 in HUVECs. (A) Cytokines were measured in HUVEC cells treated with PM2.5 (0, 40, and 80 μg·mL^−1^). IL‐4, IL‐6, IL‐8, TNF‐α, and GM‐CSF were detected, but the levels did not significantly change upon PM2.5 exposure (*n* = 3). (B) Cytokines were analyzed in the culture medium of HUVEC cells exposed to PM2.5 (0, 40, and 80 μg·mL^−1^). IL‐6, IL‐8, and GM‐CSF were detected, but only IL‐6 was secreted at a high level and significantly changed upon PM2.5 exposure (*n* = 3). *P < 0.05. Data for several cytokines (e.g., IL‐2, IL‐10, etc.) are not shown.

Previous studies have shown that inflammatory signaling increases the permeability of VECs during CVD progression, including AS 20, 21. The inflammation signaling cytokines (interleukins, lymphokines, tumor necrosis factor, etc.) involved in regulating barrier dysfunction in VECs using a high concentration of PM2.5 has not been identified. Therefore, the cell culture media and total protein of the cells from the NC and PM2.5 groups were collected, and Bio‐Plex Pro^™^ Human Cytokine 8‐plex Assay (IL‐2, IL‐4, IL‐6, IL‐8, IL‐10, TNF‐α,GM‐CSF, IFN‐g) was performed to identify inflammation‐related signaling molecules that might be affected by the high concentration of PM2.5 in VECs. The results indicated that IL‐6 was significantly elevated in the cell culture medium (Fig. 3A), but not in the cells (Fig. 3B). These complementary results suggest that high concentrations of PM2.5 initiate the inflammatory signaling pathway by promoting the secretion of IL‐6 in VECs.

### PM2.5 exposure activates hypoxia‐inducible factors

**Figure 4:**
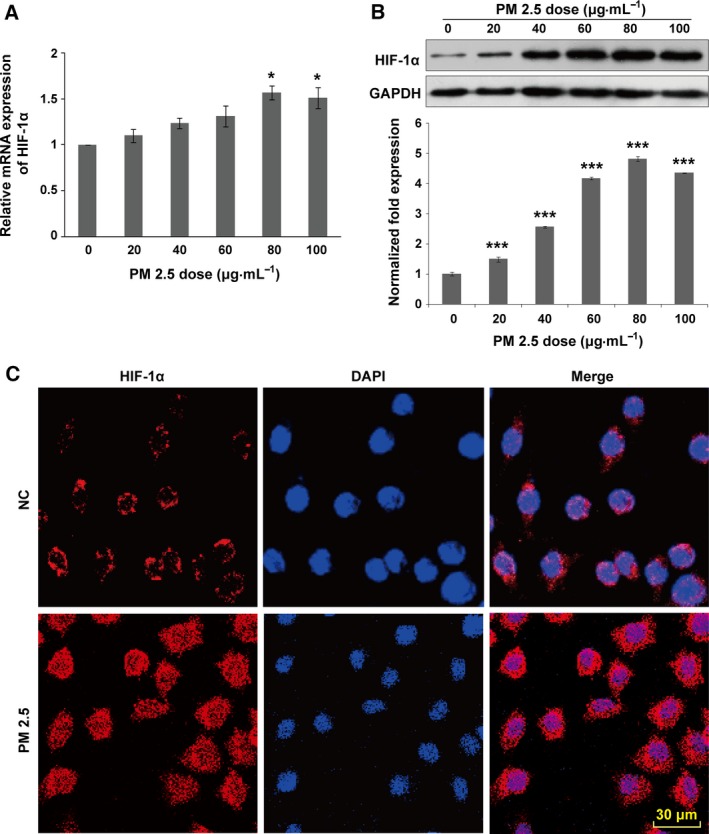
Ambient fine particulate matter increases the transcriptional activity of HIF‐1α in HUVECs. Cells were stimulated with PM2.5 at different concentrations (0, 20, 40, 60, 80, and 100 μg·mL^−1^) for 24 h. (A) PM2.5 caused a dose‐dependent increase in *HIF‐1*α mRNA expression. At a PM2.5 concentration of 80 μg·mL^−1^, *HIF‐1*α expression peaked at a level ~1.5‐fold higher than that observed in the control (*n* = 6). (B) HIF‐1α protein expression was analyzed by western blotting. The intensities of the protein bands were quantified and normalized to those of GAPDH. The data are expressed as the mean ± SD. (C) After exposure to PM2.5 (80 μg·mL^−1^) for 24 h, cells were stained for HIF‐1α by immunofluorescence. Stimulation increased the level of HIF‐1α expression and accumulated within the nucleus. Data are representative of three independent experiments performed in duplicate. Scale bar: 10 μm. *P < 0.05; ***P < 0.001. Bar: 30 μm.

Previous reports have shown that inflammatory lesions result in hypoxia, which in turn improves endothelial permeability and increases transcriptional activity of HIF‐1α, thereby promoting its expression and nuclear translocation 15, 16, 22. Moreover, an earlier investigation indicated that PM2.5 induces cardiac hypoxia and the HIF‐1α expression up‐regulation 17. However, whether PM2.5 exposure induces HIF‐1α overexpression in VECs has not been established. Therefore, total RNA was extracted from VECs treated with PM2.5 for 24 h, and quantitative RT‐PCR (qRT‐PCR) were performed to determine the level of expression of HIF‐1α. The results indicated that PM2.5 induced HIF‐1α gene expression in a dose‐dependent manner (Fig. 4A). On the other hand, the protein expression of HIF‐1α was further confirmed by western blotting, and the protein band intensities were quantified and normalized to those of GAPDH. The same induction of protein expression was observed in the cells that were exposed to PM2.5 (Fig. 4B). In addition, PM2.5 (80 μg·mL^−1^) exposure for 24 h promoted the nuclear translocation of HIF‐1α in VECs (Fig. 4C).

### Role of HIF‐1α in PM2.5‐induced IL‐6 production and cell death in HUVEC cells

**Figure 5:**
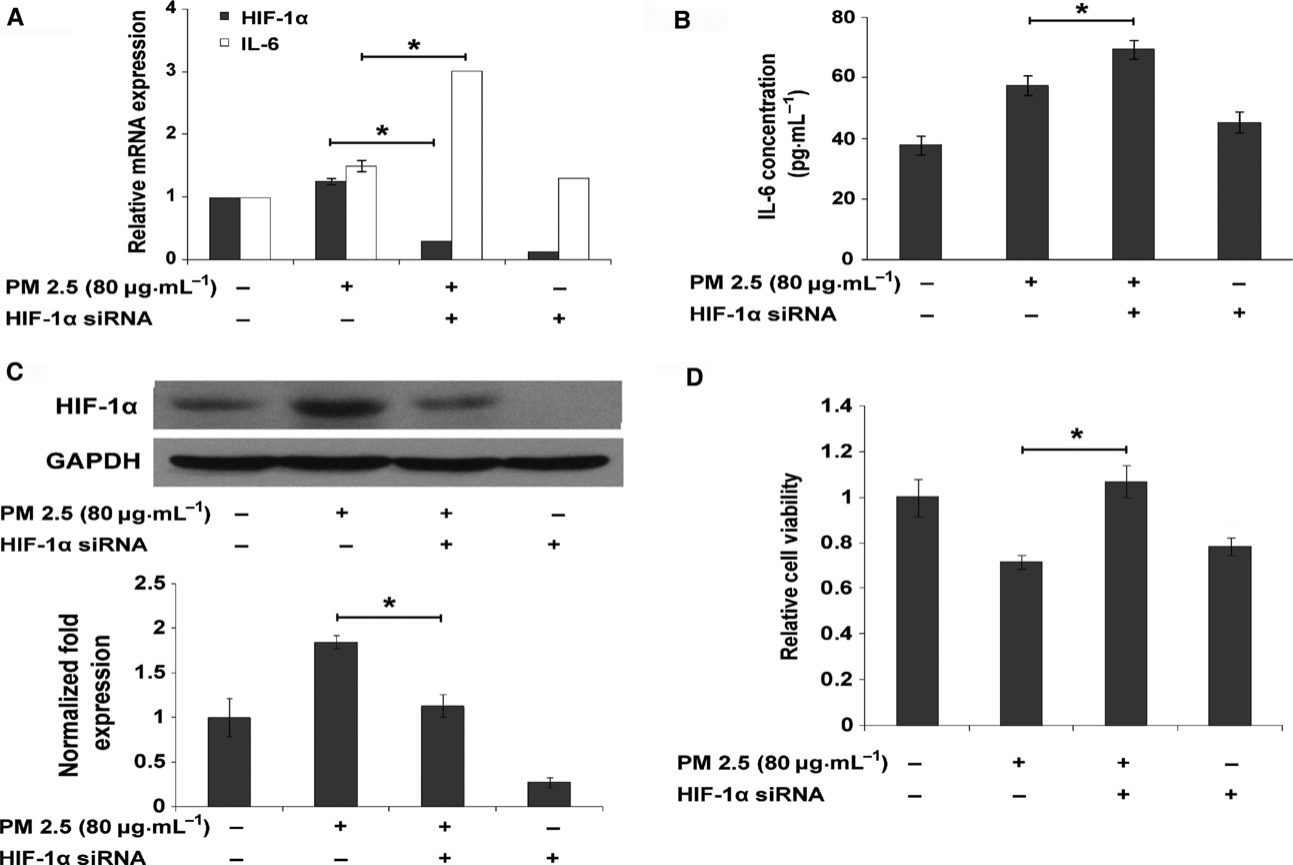
Hypoxia inducible factor‐1α‐mediated cytokine induction by PM2.5 in HUVECs. (A) *HIF‐1*α and *IL‐6* levels were analyzed by qPCR in cells treated with PM2.5 (80 μg·mL^−1^) with or without blocking HIF‐1α expression by siRNA for 12 h. Compared to the control group, *HIF‐1α* and *IL‐6* levels were both up‐regulated in the PM2.5 treatment only group. However, in cells with *HIF‐1*α down‐regulated by siRNA,*IL‐6* mRNA expression significantly increased upon PM2.5 exposure (*n* = 3). (B) IL‐6 secretion in the culture medium was detected by ELISA after HIF‐1α silencing and PM2.5 treatment. IL‐6 production increased in the PM2.5‐treated only group but was significantly up‐regulated in the siHIF‐1α/PM2.5 treatment group, compared to the other groups. Values are expressed as the mean ± SD of three independent experiments (*n* = 3). (C) HIF‐1α protein production was up‐regulated in the PM2.5 treatment only group and down‐regulated in the siHIF‐1α group. (D) After transfection with HIF‐1α siRNA, HUVECs were exposed to PM2.5 (80 μg·mL^−1^) for 24 h, and their viability was detected by CCK‐8. The viability of the siHIF‐1α group decreased by 20% compared to that of the control group and 23% versus the NC knockout group (*n* = 4, *P < 0.05).

To explore the role of HIF‐1α in PM2.5‐induced cytokine expression, HUVEC cells were transfected with HIF‐1α siRNA. Subsequently, mRNA and the protein levels of IL‐6 and HIF‐1α were analyzed. The transcript levels of *HIF‐1α* and *IL‐6* were both increased after PM2.5 stimulation; however, after HIF‐1α knockdown by siRNA, the mRNA expression of *IL‐6* sharply increased by 2.5‐fold compared with the control group (*n* = 3, *P* = 0.016) (Fig. 5A). The detected protein levels of HIF‐1α (Fig. 5C) by western blot and IL‐6 secretions (Fig. 5B) by ELISA were roughly in agreement with the gene expression analysis.

In order to understand the role of HIF‐1α in PM2.5‐induced cell death, HUVEC cells were transfected with HIF‐1α siRNA before exposure to PM2.5 (80 μg·mL^−1^) for 24 h. The viability of HUVEC cells was detected using CCK‐8. Results showed that the viability of the siHIF‐1α group decreased by 20% compared with the control group and by 23% relative to the NC knockout group (*n* = 4, *P* < 0.05) (Fig. 5D).

### PM2.5 induces barrier dysfunction in VECs by targeting IL‐6‐mediated up‐regulation of transcriptional activity of HIF‐1α

**Figure 6:**
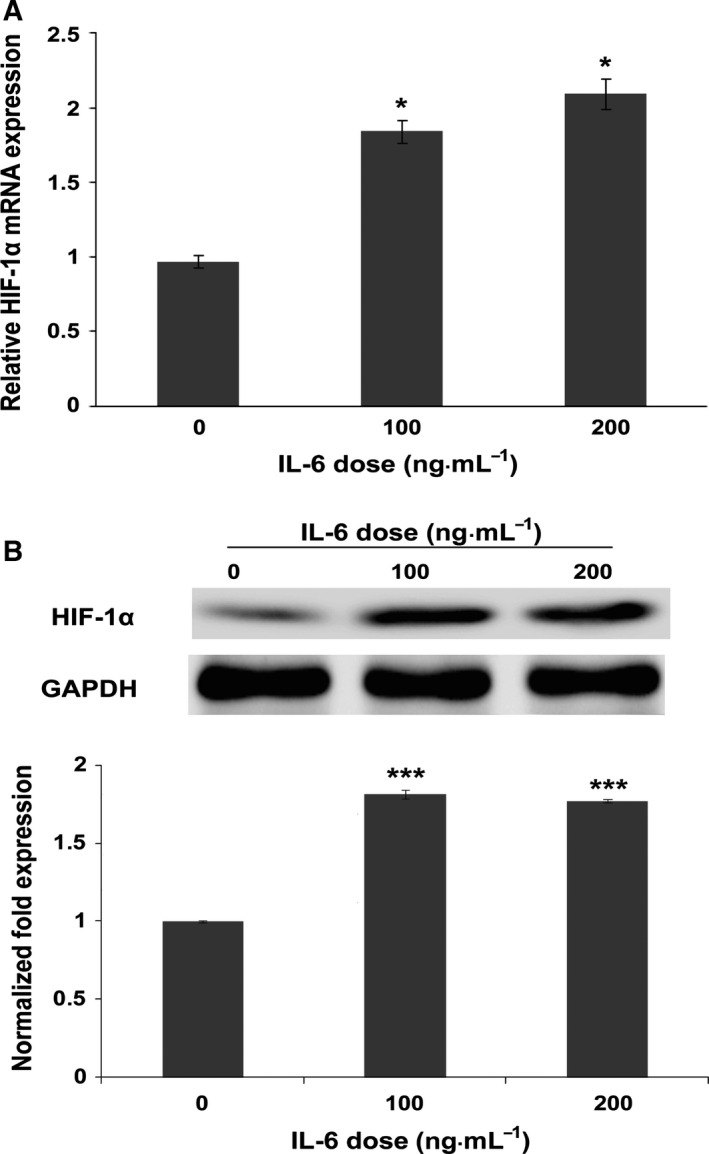
Ambient fine particulate matter induces barrier dysfunction via IL‐6/HIF‐1α signaling. HUVECs were stimulated with IL‐6 at concentrations of 0, 100 ng·mL^−1^, and 200 ng·mL^−1^ for 24 h. Total RNA and protein were then extracted for analysis. (A) *HIF‐1*α gene expression was dose dependently up‐regulated by IL‐6, with a twofold increase using a concentration of 100 ng·mL^−1^ (*n* = 3). (B) In parallel, a proportional increase in HIF‐1α protein was also observed using western blotting assay. The intensities of the protein bands were quantified and normalized to those of GAPDH. The data are expressed as the mean ± SD. *P < 0.05; ***P < 0.001.

Previous reports have indicated that hypoxia and inflammation increases endothelial permeability 12, 13, 14. However, the regulatory mechanism between inflammation and hypoxia in high concentrations of PM2.5 in relation to barrier dysfunction in VECs remains elusive. To address this question, the effect of IL‐6 on the expression of HIF‐1α in VECs was analyzed. VECs treated with the indicated concentration of IL‐6 for 24 h resulted in a marked increase in HIF‐1α transcription (Fig. 6A). Moreover, a similar up‐regulation in HIF‐1α protein was observed in IL‐6 treated VECs (Fig. 6B). Taken together, our data demonstrated that HIF‐1α might be a key mediator of IL‐6 in the regulation of endothelium permeability during exposure to high concentrations of PM2.5.

## Discussion

This study has clearly demonstrated the role of particulate matter (PM2.5) on vascular endothelial cells (VECs) barrier function. Our findings support a model that exposure to high concentrations of PM2.5 directly results in an increase in the secretion of IL‐6, which then up‐regulates the expression and promotes nuclear translocation of HIF‐1α, thereby suppressing cell proliferation and increasing membrane permeability in VEC.

Several previous reports have indicated that the toxicity of PM2.5 is directly related to its composition 23, 24, 25. Therefore, most of the effects associated with PM2.5 in the present study are likely due to its main components, namely, heavy metals and PAHs (5, 6). One of main components of PM2.5 from Beijing, PAH (5, 6), which is mainly produced from automobile exhaust fumes, can cause cardiovascular events 26. However, its effect on early vascular cells (EVCs) involved in systemic circulation remains elusive. Our study demonstrated that concentrated PM2.5, which includes a high content of heavy metals and PAHs, significantly increased membrane permeability. However, further studies examining its contribution to barrier dysfunction in EVCs are warranted.

Although numerous studies have indicated that PM2.5 can destroy the cardiovascular system thus resulting in morbidity and mortality in relation to cardiovascular disease (CVD), its underlying mechanisms remain largely unknown. Previous studies have demonstrated that PM2.5 induces endothelial dysfunction in pulmonary arteries by triggering local inflammation, followed by the secretion of proinflammatory cytokines that further mediate advanced cardiovascular events 27. It has also been indicated that acute and long‐term exposure to PM2.5 induces endothelial dysfunction in systemic arteries 27, 28, 29. Nevertheless, the direct effect of PM2.5 on VECs is still unknown. Due to its small aerodynamic diameter, PM2.5 could diffuse into the blood circulation from the respiratory system and directly come into contact with VECs, which is the innermost layer of the blood vessels. Therefore, PM2.5 may pose a direct threat to VEC barrier function. Our study has confirmed that PM2.5 can directly destroy the membrane barrier function of VECs upon entry into the blood circulation. As previously reported, endothelial permeability is critical for the formation and development of AS 9, 10, 11.

Numerous reports have demonstrated that PM2.5 exposure causes barrier dysfunction of the pulmonary capillary bed by inducing the expression of inflammatory cytokines 27. Moreover, this inflammatory response could increase membrane permeability by promoting ROS/TRPM2/Calpain/ZO‐1 signaling, which is PM‐activated reactive oxygen species (ROS), transient receptor potential channels (*TRPM2)* promotes zonula occludens‐1 *(ZO‐1)* degradation by calcium‐activated neutral protease *(calpain)* or alternating the expression of endothelial activation factors 30. However, the regulatory mechanism of inflammatory cytokines that are involved in membrane permeability has not been established. In fact, PM2.5 has been widely demonstrated to induce cytokine secretions upon exposure to epithelial cells *in vitro*. IL‐6 could cause cell membrane damage and induce endothelial cell contraction 31 by affecting myosin light chain kinase (MLCK) 14. Cellular adaptations to hypoxia processes depend on the transcription factor, HIF‐1α, which is often degraded and located in the cytoplasm under normoxic conditions, but its stability and transcriptional activity is significantly increased during hypoxia 22. In the present study, we demonstrated that concentrated PM2.5 promotes inflammation and increases the expression of IL‐6, which then activates the transcriptional activity of HIF‐1 in VECs. Altogether, the functional and molecular alterations observed in VECs may provide a new evidence that elucidates the mechanism underlying the development of CVDs in response to PM2.5 exposure.

In summary, our findings have demonstrated that PM2.5 is critically involved in the endothelial permeability in systemic blood vessels and may serve as a potential threat to cardiovascular disease.

## References

[1] Brook RD and Rajagopalan S (2010) Particulate matter air pollution and atherosclerosis. Curr Atheroscler Rep 12, 291–300. 20617466 10.1007/s11883-010-0122-7

[2] Brook RD , Franklin B , Cascio W , Hong Y , Howard G , Lipsett M , Luepker R , Mittleman M , Samet J , Smith SC Jr *et al* (2004) Air pollution and cardiovascular disease: a statement for healthcare professionals from the Expert Panel on Population and Prevention Science of the American Heart Association. Circulation 109, 2655–2671. 15173049 10.1161/01.CIR.0000128587.30041.C8

[3] Wan Q , Cui X , Shao J , Zhou F , Jia Y , Sun X , Zhao X , Chen Y , Diao J and Zhang L (2014) Beijing ambient particle exposure accelerates atherosclerosis in ApoE knockout mice by upregulating visfatin expression. Cell Stress Chaperones 19, 715–724. 24523034 10.1007/s12192-014-0499-2 PMC4147068

[4] Mills NL , Donaldson K , Hadoke PW , Boon NA , MacNee W , Cassee FR , Sandström T , Blomberg A and Newby DE (2009) Adverse cardiovascular effects of air pollution. Nat Clin Pract Cardiovasc Med 6, 36–44. 19029991 10.1038/ncpcardio1399

[5] Dockery DW and Stone PH (2007) Cardiovascular risks from fine particulate air pollution. N Engl J Med 356, 511–513. 17267912 10.1056/NEJMe068274

[6] Sun Q , Wang A , Jin X , Natanzon A , Duquaine D , Brook RD , Aguinaldo JG , Fayad ZA , Fuster V , Lippmann M *et al* (2005) Long‐term air pollution exposure and acceleration of atherosclerosis and vascular inflammation in an animal model. JAMA 294, 3003–3010. 16414948 10.1001/jama.294.23.3003

[7] Tamagawa E , Bai N , Morimoto K , Gray C , Mui T , Yatera K , Zhang X , Xing L , Li Y , Laher I *et al* (2008) Particulate matter exposure induces persistent lung inflammation and endothelial dysfunction. Am J Physiol Lung Cell Mol Physiol 295, L79–L85. 18469117 10.1152/ajplung.00048.2007 PMC2494798

[8] Zhang WC , Wang YG , Zhu ZF , Wu FQ , Peng YD , Chen ZY , Yang JH , Wu JJ , Lian YT , He MA *et al* (2014) Regulatory T cells protect fine particulate matter‐induced inflammatory responses in human umbilical vein endothelial cells. Mediators Inflamm 2014, 869148. 24987196 10.1155/2014/869148 PMC4060066

[9] Sima AV , Stancu CS and Simionescu M (2009) Vascular endothelium in atherosclerosis. Cell Tissue Res 335, 191–203. 18797930 10.1007/s00441-008-0678-5

[10] Cullen P , Rauterberg J and Lorkowski S (2005) The pathogenesis of atherosclerosis. Handb Exp Pharmacol 10, 3–70. 16596795 10.1007/3-540-27661-0_1

[11] Phinikaridou A , Andia ME , Protti A , Indermuehle A , Shah A , Smith A , Warley A and Botnar RM (2012) Noninvasive magnetic resonance imaging evaluation of endothelial permeability in murine atherosclerosis using an albumin‐binding contrast agent. Circulation 126, 707–719. 22753191 10.1161/CIRCULATIONAHA.112.092098

[12] van den Oever IA , Raterman HG , Nurmohamed MT and Simsek S (2010) Endothelial dysfunction, inflammation, and apoptosis in diabetes mellitus. Mediators Inflamm 2010, 792393. 20634940 10.1155/2010/792393 PMC2903979

[13] Park JH , Okayama N , Gute D , Krsmanovic A , Battarbee H and Alexander JS (1999) Hypoxia/aglycemia increases endothelial permeability: role of second messengers and cytoskeleton. Am J Physiol 277, C1066–C1074. 10600758 10.1152/ajpcell.1999.277.6.C1066

[14] Maruo N , Morita I , Shirao M and Murota S (1992) IL‐6 increases endothelial permeability in vitro. Endocrinology 131, 710–714. 1639018 10.1210/endo.131.2.1639018

[15] Koeppen M , Eckle T and Eltzschig HK (2011) The hypoxia‐inflammation link and potential drug targets. Curr Opin Anaesthesiol 24, 363–369. 21659868 10.1097/ACO.0b013e32834873fd PMC3259566

[16] Giatromanolaki A , Sivridis E , Maltezos E , Papazoglou D , Simopoulos C , Gatter KC , Harris AL and Koukourakis MI (2003) Hypoxia inducible factor 1alpha and 2alpha overexpression in inflammatory bowel disease. J Clin Pathol 56, 209–213. 12610101 10.1136/jcp.56.3.209 PMC1769899

[17] Rivero DH , Soares SR , Lorenzi‐Filho G , Saiki M , Godleski JJ , Antonangelo L , Dolhnikoff M and Saldiva PH (2005) Acute cardiopulmonary alterations induced by fine particulate matter of Sao Paulo, Brazil. Toxicol Sci 85, 898–905. 15746007 10.1093/toxsci/kfi137

[18] Zhang X , Kon T , Wang H , Li F , Huang Q , Rabbani ZN , Kirkpatrick JP , Vujaskovic Z , Dewhirst MW and Li CY (2004) Enhancement of hypoxia‐induced tumor cell death in vitro and radiation therapy in vivo by use of small interfering RNA targeted to hypoxia‐inducible factor‐1alpha. Cancer Res 64, 8139–8142. 15548675 10.1158/0008-5472.CAN-03-2301

[19] Wei Y , Han IK , Hu M , Shao M , Zhang JJ and Tang X (2010) Personal exposure to particulate PAHs and anthraquinone and oxidative DNA damages in humans. Chemosphere 81, 1280–1285. 20869742 10.1016/j.chemosphere.2010.08.055

[20] Riccioni G , Zanasi A , Vitulano N , Mancini B and D'Orazio N (2009) Leukotrienes in atherosclerosis: new target insights and future therapy perspectives. Mediators Inflamm 2009, 737282. 20150962 10.1155/2009/737282 PMC2817543

[21] Harja E , Bu DX , Hudson BI , Chang JS , Shen X , Hallam K , Kalea AZ , Lu Y , Rosario RH , Oruganti S *et al* (2008) Vascular and inflammatory stresses mediate atherosclerosis via RAGE and its ligands in apoE‐/‐ mice. J Clin Invest 118, 183–194. 18079965 10.1172/JCI32703 PMC2129235

[22] Eltzschig HK and Carmeliet P (2011) Hypoxia and inflammation. N Engl J Med 364, 656–665. 21323543 10.1056/NEJMra0910283 PMC3930928

[23] Hopke PK and Rossner A (2006) Exposure to airborne particulate matter in the ambient, indoor, and occupational environments. Clin Occup Environ Med 5, 747–771. 17110290 10.1016/j.coem.2006.08.001

[24] Shi J , Shao W , Yang D , Zhao L , Deng L , Wang X and Sun B (2010) Hydrodynamics‐based transfection of plasmid encoding receptor activator for nuclear factor kappa B‐Fc protects against hepatic ischemia/reperfusion injury in mice. Liver Transpl 16, 611–620. 20440770 10.1002/lt.22030

[25] Adamson IY , Prieditis H , Hedgecock C and Vincent R (2000) Zinc is the toxic factor in the lung response to an atmospheric particulate sample. Toxicol Appl Pharmacol 166, 111–119. 10896852 10.1006/taap.2000.8955

[26] Laszt L and Schaad R (1973) Experimental in‐vivo and in‐vitro studies on the effect of automobile exhaust and carbon monoxide on the cardiovascular system of mammals. Basic Res Cardiol 68, 380–394. 4125896 10.1007/BF01906175

[27] Davel AP , Lemos M , Pastro LM , Pedro SC , deAndré PA , Hebeda C , Farsky SH , Saldiva PH and Rossoni LV (2012) Endothelial dysfunction in the pulmonary artery induced by concentrated fine particulate matter exposure is associated with local but not systemic inflammation. Toxicology 295, 39–46. 22361244 10.1016/j.tox.2012.02.004

[28] Nurkiewicz TR , Porter DW , Barger M , Millecchia L , Rao KM , Marvar PJ , Hubbs AF , Castranova V and Boegehold MA (2006) Systemic microvascular dysfunction and inflammation after pulmonary particulate matter exposure. Environ Health Perspect 114, 412–419. 16507465 10.1289/ehp.8413 PMC1392236

[29] Kampfrath T , Maiseyeu A , Ying Z , Shah Z , Deiuliis JA , Xu X , Kherada N , Brook RD , Reddy KM , Padture NP *et al* (2011) Chronic fine particulate matter exposure induces systemic vascular dysfunction via NADPH oxidase and TLR4 pathways. Circ Res 108, 716–726. 21273555 10.1161/CIRCRESAHA.110.237560 PMC3085907

[30] Wang T , Wang L , Moreno‐Vinasco L , Lang GD , Siegler JH , Mathew B , Usatyuk PV , Samet JM , Geyh AS , Breysse PN *et al* (2012) Particulate matter air pollution disrupts endothelial cell barrier via calpain‐mediated tight junction protein degradation. Part Fibre Toxicol 9, 35. 22931549 10.1186/1743-8977-9-35 PMC3489700

[31] Yamauchi‐Takihara K , Ihara Y , Ogata A , Yoshizaki K , Azuma J and Kishimoto T (1995) Hypoxic stress induces cardiac myocyte‐derived interleukin‐6. Circulation 91, 1520–1524. 7867193 10.1161/01.cir.91.5.1520

